# A Study on the Prevalence of Occupational Stress Among Police Personnel in Delhi

**DOI:** 10.7759/cureus.86588

**Published:** 2025-06-23

**Authors:** Neha Yadav, Geeta Yadav

**Affiliations:** 1 Community Medicine, University College of Medical Sciences, Delhi, IND; 2 Community Medicine, Vardhman Mahavir Medical College and Safdarjung Hospital, Delhi, IND

**Keywords:** india, occupational stress, operational stress, organisational stress, police, stressors

## Abstract

Introduction

Occupational stress or stress at work is a real challenge for workers as well as organisations. Work-related stress can be caused by poor work organisation, poor work design, poor management, unsatisfactory working conditions, and lack of support from colleagues and supervisors. Police personnel work in such conditions that make them vulnerable to adverse physiological and psychological outcomes; however, this often goes underreported, underdiagnosed, and undertreated. The current study focused on two types of occupational stress, namely operational stress and organisational stress. Operational stress is due to the nature of the job, whereas organisational stress is due to the organisational culture in which a person works in.

Aims and objectives

The aim of the study was to assess the prevalence of occupational stress and associated factors among police personnel in Delhi.

Methodology

This cross-sectional study was conducted among 374 police personnel in a selected district in Delhi. The district was selected randomly via the chit method, and in the second stage, a non-proportional stratified random sampling technique was used, and two strata were constituted of 187 participants in each stratum. The first stratum included inspectors, sub-inspectors (SI), and assistant sub-inspectors (ASI), and the second stratum included constables and head-constables. For the assessment of occupational stress, the Police Stress Questionnaire (PSQ) was used.

Results

The prevalence of stress (moderate to high) was found to be 91.5% and 54.2% for operational and organizational stress. Females (17.6% of the study participants) were found to be more stressed. Not having enough time available to spend with friends and family, and staff shortages were found to be the most stressful operational and organisational stressors, respectively.

Conclusion

The study revealed a high prevalence of occupational stress among police personnel, highlighting the urgent need for intervention and proactive measures by relevant authorities.

## Introduction

Worldwide, work stress is recognized as a major challenge to workers' health and the healthiness of their organisations [1]. Occupational or job-related stress refers to a situation wherein job-related factors interact with the worker to change his/her psychological/physiological condition, such that the person is forced to deviate from normal functioning [2]. Simply put, it is the response people may have when presented with work demands and pressures that are not matched to their knowledge and abilities and challenge their ability to cope [1]. 

Policing is a stressful occupation as it is one of the few jobs where employees are expected to face physical dangers and risk their lives [3]. Police personnel working in such conditions are vulnerable to adverse physiological and psychological outcomes [4]. However, this often goes underreported, underdiagnosed, and undertreated, thus affecting the quality of the workforce. Few studies that have been conducted in India (in different places) using different scales to measure stress among police personnel have reported occupational stress varying from 25% to as high as 90% [5-10]. The current study focused on two types of occupational stress, namely organisational stress and operational stress. Organisational stress arises from the culture and structure of the organisation in which an individual works. It is typically caused by factors such as staff shortages, inadequate resources, excessive responsibilities, bureaucratic hurdles (red tapism), and inefficient management practices [11]. In contrast, operational stress stems from the inherent nature of the job itself. It includes challenges like extended working hours, physical and mental fatigue, the risk of injury, limited time for personal or social life, and the constant pressure to perform under demanding conditions [11]. 

The current study was conducted in a city, Delhi, which has a commissionerate system of policing. The structural organization of police in a commissionerate system in India at the district level has the following hierarchical arrangement. Constabulary (including constables and head constables) forms the lowest tier, and their main job is to follow the orders of their seniors. They are headed by Inspectors, sub-inspectors (SI), and assistant sub-inspectors (ASI) who are largely responsible for investigation and law and order situation management in their jurisdiction and supervision of the police stations. They are headed by the assistant commissioner of police (ACP), the deputy commissioner of police, and the additional deputy commissioner of police.

There is a paucity of literature on occupational stress among police personnel in Delhi, which, being the capital of the country, poses unique challenges and threats. This study is a step in the direction of providing meaningful input for a better understanding of occupational stress among police personnel and can prove to nudge the police organisational, operational structure, and work culture, help in better policy making, and ensure better policing and a safe environment for all.

## Materials and methods

An observational, descriptive cross-sectional study was conducted among 374 police personnel from among the 1063 police personnel posted in a district of Delhi. This study was conducted from November 2020 to April 2022. All the police personnel posted in the selected district and available during the time of the study were included in the study. There was no exclusion criteria. 

Sample size

Based on the prevalence rates of moderate operational stress (67%) and moderate organisational stress (68%) as reported by Vivek et al. (2018), the sample size was calculated using the formula (Zα/2)2PQ/L2. This yielded sample sizes of 340 for operational stress and 335 for organisational stress. The higher value of 340 was chosen as the base sample size. To account for a 10% non-response error rate, the final adjusted sample size was calculated to be 374. 

Sampling technique

A two-stage random sampling method was employed for the present study. In the first stage, one district was selected from among the 15 districts in Delhi using simple random sampling by the Chit method. In the second stage, a non-proportional stratified random sampling technique was applied, and two strata were created, stratum 1 and stratum 2, with 187 participants in each of them. Stratum 1 included inspectors, sub-inspectors (SI), and assistant sub-inspectors (ASI), and stratum 2 included constables and head constables. At the time of the study, 1063 police personnel were posted in the selected district. From these, the first 184 participants in each stratum who consented to participate in the study were selected. All the police personnel in the district from all designations, including those involved in law and order management, criminal investigations, administrative duties, and other roles, were included in the study, except for officers from the Indian Police Service (IPS). 

Study tool

The study used a self-administered Police Stress Questionnaire (PSQ) for the assessment of occupational stress and a pretested semi-open-ended interviewer-administered bilingual questionnaire to assess socio-demographic characteristics, occupation-related details, etc [11]. The PSQ, consists of two questionnaires, Organizational Police Stress Questionnaire (PSQ-Org) and Operational Police Stress Questionnaire (PSQ-Op), containing 20 questions each. These are validated questionnaires that have been used in various Indian studies, including studies by Vivek et al. (2018) and Ragesh et al.(2017)[8-9].

Responses to PSQ were taken for each item on a seven-point Likert scale that ranges from no stress at all (1) to a lot of stress (7).

Outcome measures

For operational stress, the mean total cut-off score for low, moderate, and high stress was taken at below 2.0, 2.1-3.4, and above 3.5, respectively [12]. For organizational stress, the mean total cut-off score for low, moderate, and high stress was taken at below 2.6, 2.7-3.9, and above 4.0, respectively [12]. For blood pressure, the cut-off was taken as >140/90 mmHg. The BMI categorisation that was taken for the present study is underweight (BMI <18.5), normal (BMI 18.5-22.99), overweight (BMI 23.0-24.99), and obese (BMI> 25) [13]. 

Data analysis

Quantitative variables were depicted with mean, standard deviation, median, interquartile range (IQR), etc. Qualitative variables were expressed in proportion. An appropriate diagrammatic representation was used to represent the data. The association was tested by the Chi-squared (χ^2^) test/ Fisher's exact test. A t-test was used to compare means. A p-value less than 0.05 was taken as significant. All p-values in the study were calculated using two-tailed tests. Data analysis was done using SPSS (IBM, Armonk, New York) software, licensed version 21.0.

Ethical consideration

Institutional Review Board (IRB) and Institutional Ethics Committee of Vardhman Mahavir Medical College and Safdarjung Hospital, New Delhi (Approval number IEC/VMMC/SJH/Thesis/2020-11/CC-80) approved the study, and informed consent was obtained from the participants. Permission was also taken from the concerned authority for the selected district prior to the study.

## Results

The study sample consisted of 374 police personnel. The mean age of the study participants was 41.02 years (with standard deviation ±9.81) and the majority of the study participants were males (82.4%), married (93.9%), Hindu (97.9%), living in a joint family (64.2%), graduate/postgraduate (68.4%), and belonged to upper-class socio-economic status according to the modified Brahm Govind Prasad scale (89%; Table 1). Most of the study participants were in service for more than 10 years (68.7%), reported to have less than four holidays per month (79.9%), and worked for more than 48 hours per week (68.5%; Table 1).

**Table 1:** Sociodemographic and occupational characteristics of study participants (N=374)

Age group* (in years)	Number (%)
21-30	55 (14.7)
31-40	141 (37.7)
41-50	86 (23.0)
51-60	92 (24.6)
*Mean age (years) = 41.02 (± 9.81)	
Sex	
Male	308 (82.4)
Female	66 (17.6)
Marital status	
Married	351 (93.9)
Unmarried/Divorced/ Separated/ Widowed	23 (6.15.6)
Family type	
Nuclear	134 (35.8)
Joint	240 (64.2)
Educational qualification	
10th Pass	29 (7.8)
12th Pass	89 (23.8)
Graduate and above	256 (68.4)
Socio-economic status (according to modified Brahm Govind Prasad scale)	
Upper class	333 (89.0)
Upper-middle-class and middle class	41 (11.0)
Duration of service (in years)	
<10 years	117 (31.3)
>10 years	257 (68.7)
Duty hours/week	
<48 hours	118 (31.5)
49-72 hours	182 (48.7)
>72 hours	74 (19.8)

**Figure 1:**
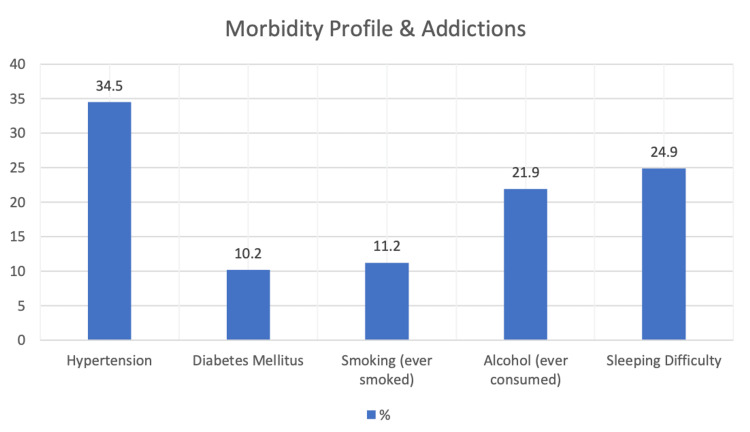
Distribution of study participants according to medical and personal history (N=374)

Out of a total of 374 participants, hypertension was present in 51 (13.6%) and diabetes mellitus in 38 (10.2%) participants. Almost 22% and 11.2% of study participants reported having consumed alcohol and smoked tobacco at some point in their lives, respectively, and 18.4% and 8.3% of the study participants are current alcohol consumers and smokers. Around one-fourth (24.9%) of the participants reported difficulty in sleeping (Figure 1).

**Figure 2:**
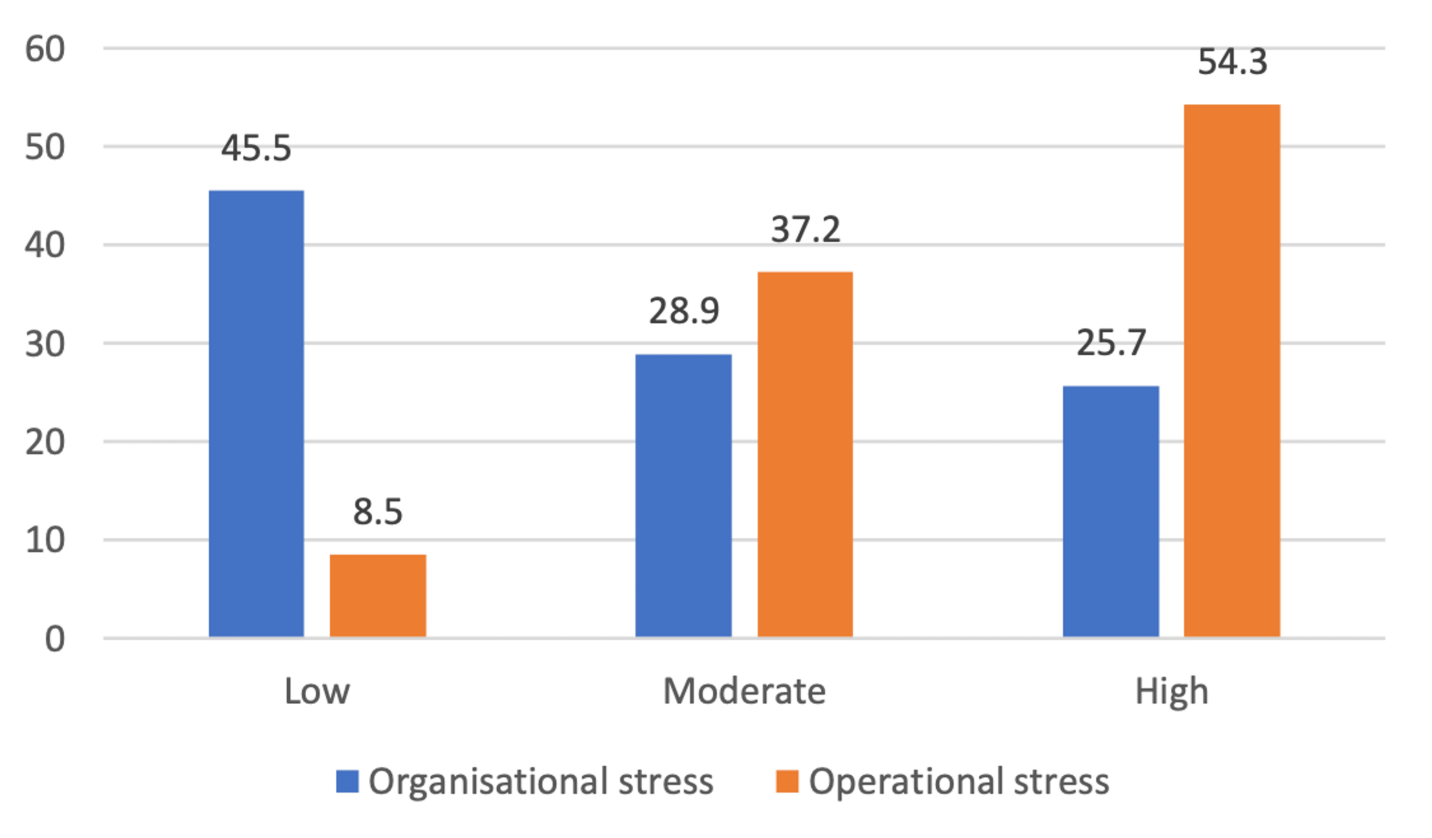
Prevalence of organisational and operational stress among study participants (N=374)

**Figure 3:**
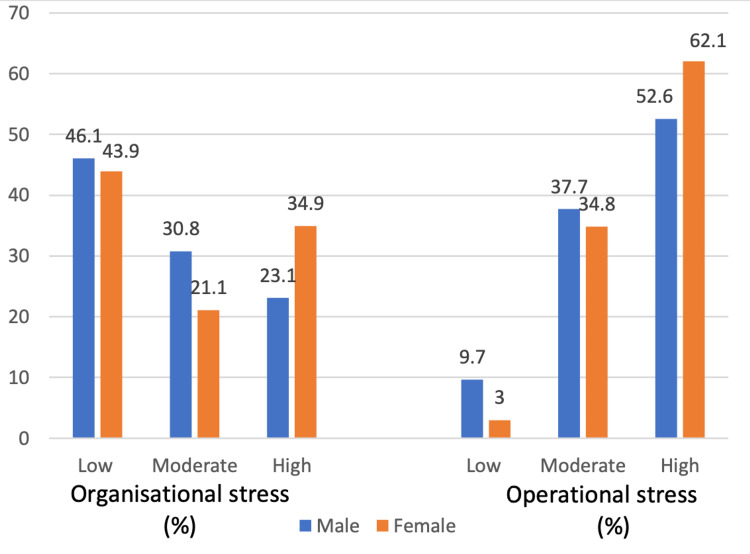
Distribution of occupational stress (organisational and operational) among study participants according to gender (N=374)

On analysing the organisational stress, it was found that 29.1% experienced moderate stress and 25.1% experienced high stress, whereas, in the case of operational stress, moderate stress was reported by 37.2% and high stress by 54.3% (Figure 2). The prevalence was higher among female participants than their male counterparts (56.1% vs 53.9% for organisational stress and 96.9% vs 90.3% for operational stress; Figure 3).

Among the various causes of organizational stress, staff shortages were perceived to be as most stressful (mean +SD=4.16±2.01) followed by a lack of resources (3.53±2.52), Excessive administrative duties (3.52±2.21), Unequal sharing of work (3.52±2.21), lack of training (3.41±2.17) and feeling accountable for doing your job (3.34±2.23) respectively. Male participants perceived staff shortage (4.26±2.31), followed by lack of resources (3.54±2.20), as most stressful, whereas female participants perceived excessive administrative duties (4.27±2.05) and staff shortage (3.71±2.18) as most stressful (Table 2). Among all the operational stressors, not having enough time available to spend with friends and family was found to be the most stressful (mean±SD=4.62±1.97), followed by managing social life outside work (4.50±1.98, finding time to stay in good physical condition (4.49±2.07), fatigue (4.46±1.93), feeling like always being on job (4.28±2.137). Male participants perceived not having enough time to spend with friends and family as the most stressful, with a mean score of 4.56+1.98, while female participants perceived managing social life outside work with a mean score of 5.06+1.83 as the most stressful (Table 3).

**Table 2:** Mean scores for various organizational stressors as per PSQ-Org among study participants (N=374) PSQ-Org - Organizational Police Stress Questionnaire

Organisational stressors	Male (n=308) (mean±SD)	Female (n=66) (mean±SD)	Overall (mean±SD)
Dealing with co-workers	1.86±1.45	3.55±1.97	2.16±1.68
Feeling that different rules apply to different people	3.09±2.00	3.27±2.38	3.12±2.07
To prove yourself to the organisation	2.99±2.06	3.73±2.33	3.12±2.12
Excessive administrative duties	3.36±2.04	4.27±2.05	3.52±2.07
Constant changes in policy/legislation	2.97±1.98)	3.17±2.15	3.01±2.01
Staff shortages	4.26±2.31	3.71±2.18	4.16±2.01
Bureaucratic red tape	3.32±2.29	3.00±2.01	3.26±2.25
Too much computer work	2.97±2.09	3.65±2.13	3.09±2.11
Lack of training on new equipment	3.44±2.15	3.29±2.27	3.41±2.17
Perceived pressure to volunteer free time	2.23±1.70	2.86±1.70	2.34±1.71
Dealing with supervisors	2.22±1.82	2.38±1.65	2.25±1.79
Inconsistent leadership style	2.67±2.02	2.62±1.80	2.66±1.98
Lack of resources	3.54±2.20	3.50±2.48	3.53±2.52
Unequal sharing of work responsibilities	3.52±2.19	3.55±2.35	3.52±2.21
Looked down on by co-workers when sick or injured	1.79±1.45	2.47±2.03	1.91±1.58
Over-emphasis on the negatives by leaders	2.60±1.96	2.88±1.93	2.65±1.95
Internal investigations	2.66±2.09	2.53±2.12	2.64±2.09
Dealing with the court system	3.06±2.28	2.79±2.20	3.01±2.27
Need to be accountable for doing the job	3.30±2.22	3.52±2.30	3.34±2.23
Inadequate equipment	3.11±2.14	3.74±2.30	3.22±2.18

**Table 3:** Mean scores for various stressors for operational stress as per PSQ-Op among study participants (N=374) PSQ-Op - Operational Police Stress Questionnaire

Operational stressors	Male (n=308)	Female (n=66)	Overall score
Shift work	2.30±1.73	2.70±2.05	2.37±1.80
Working alone at night	3.29±2.25	3.89±2.33	3.40±2.27
Over-time demands	3.77±2.15	3.86±2.25	3.79±2.17
Risk of being injured on the job	3.88±2.31	3.53±2.20	3.82±2.29
Work-related activities on day soff	3.89±2.18	3.02±2.16	3.74±2.20
Traumatic events	3.17±2.30	3.45±2.26	3.22±2.29
Managing social life outside of work	4.38±1.99	5.06±1.83	4.50±1.99
Not enough time available to spend with friends and family	4.56±1.99	4.94±1.83	4.62±1.97
Paperwork	3.69±1.68	4.15±1.70	3.77±1.69
Eating healthy at work	3.91±2.21	4.48±2.08	4.01±2.19
Finding time to stay in good physical condition	4.46±2.11	4.64±1.91	4.49±2.08
Fatigue (e.g., shift work, overtime)	4.35±1.94	4.95±1.84	4.46±1.93
Occupation-related health issues	3.88±2.30	5.08±2.22	4.09±2.33
Lack of understanding from family and friends about work	3.31±2.10	3.42±2.05	3.33±2.09
Making friends outside the job	2.85±1.96	3.17±2.26	2.91±2.01
Maintaining a higher image in public	3.62±2.07	4.11±2.21	3.71±2.10
Negative comments from the public	4.12±2.30	3.94±2.16	4.09±2.27
Limitations to social life	3.89±2.15	4.06±1.61	3.92±2.07
Feeling like always being on the job	4.23±2.19	4.48±1.86	4.28±2.14
Friends/family feel the effects of the stigma associated with the job	3.51±2.22	4.17±2.08	3.62±2.21

The study found a statistically significant association between type of family (p=0.01), educational status (p=0.009), and organizational stress. The prevalence of organizational stress (moderate and high level) was found to be higher in participants living in a joint family (n=142, 59.2%) and who were 10th class (secondary school certificate or high school) pass (n=22, 75.9%) or graduate and above (n=142, 55.5%). The study found a statistically significant association between organisational stress and years of service and duty hours per week. Those in service for more than 10 years and working for more than 72 hours per week had more organisational stress (Table 4). A statistically significant association was observed between operational stress (moderate and high) and education status (p=0.001), and socio-economic status (p=0.015). Higher levels of operational stress were seen in participants belonging to a higher socio-economic class. No statistically significant association was observed between operational stress and type of family, years of service (p=0.423), or duty hours per week (p=0.448; Table 5). No statistically significant association was found between occupational stress (organisational as well as operational stress), and age of the participants, gender, marital status, rank strata, and holidays in a month (Tables 4, 5).

**Table 4:** Association between organizational stress and various factors (N=374) * statistically significant p-value

Factors	Organizational stress			χ^2^ value (p-value)*
	Moderate and above n (%)	Low n (%)	Total n (%)	
Age (years)				5.036 (0.16)
21-30	30 (54.5)	25 (45.5)	55 (14.7)	
31-40	67 (47.5)	74 (52.5)	141 (3.7)	
41-50	49 (57.0)	37 (43.0)	86 (23)	
51-60	57 (62.0)	35 (38.0)	92 (24.6)	
Gender				
Male	166 (53.9)	142 (46.1)	308 (82.3)	0.103 (0.74)
Female	37 (56.1)	29 (43.9)	66 (17.6)	
Marital status				
Married	89 (52.8)	162 (46.2)	351 (93.9)	0.429 (0.51)
Single /divorced/widowed	14 (60.9)	9 (39.1)	23 (6.1)	
Family type				
Nuclear	61 (45.5)	73 (54.5)	333 (89)	6.451(0.01)*
Joint	142 (59.2)	98 (40.8)	41 (11)	
Education status				
10th pass	22 (75.9)	7 (24.1)	29 (7.8)	9.512 (0.009)*
12th pass	39 (43.8)	50 (56.2)	89 (23.8)	
Graduate and above	142 (55.5)	114 (44.5)	256 (68.4)	
Socio-economic status				
Upper class	181 (54.4)	152 (45.6)	333 (89)	0.007 (0.93)
Upper-middle/ middle class	22 (53.7)	19 (46.3)	41 (11)	
Rank strata				
Rank strata 1	109 (58.3)	78 (41.7)	187 (100)	2.42 (0.11)
Rank strata 2	94 (50.3)	93 (49.7)	187 (100)	
Years of service				
<10 years	51 (43.6)	66 (56.4)	117 (100)	7.83 (0.005)*
>10 years	152 (59.1)	105 (40.9)	257( 100)	
Duty hours				
≤ 48	66 (55.9)	52 (44)	118 (100)	13.94 (0.001)*
49-72	84 (46.2)	98 (53.8)	182 (100)	
>72	53 (71.6)	21 (28.4)	74 (100)	
Holidays in a month				
<4 days	158 (52.8)	141 (47.2)	299 (100)	1.238 (0.26)
>4 days	45 (60)	30 (40)	75 (100)	

**Table 5:** Association between operational stress and various factors (N=374) *p-value for Chi^2^ test **Fishers Exact Test

Factors	Operational stress			X^2^ value (p-value)*
	Moderate and above n (%)	Low n (%)	Total n (%)	
Age (years)				
21-30	52 (94.5)	3 (5.5)	55 (100)	4.79 (0.187)
31-40	128 (90.8)	13 (9.2)	141 (100)	
41-50	82 (95.3)	4 (4.7)	86 (100)	
51-60	80 (87)	12 (13)	92 (100)	
Gender				
Male	278 (90.3)	30 (9.7)	308 (100)	3.12(0.07)
Female	64 (97)	2 (3)	66 (100)	
Marital status				
Married	319 (90.9)	32 (9.1)	351 (100)	2.29 (0.13)
Single/divorced/widowed	23 (100)	0	23 (100)	
Family type				
Nuclear	118 (88.1)	16 (11.9)	134 (100)	3.05 (0.08)
Joint	224 (93.3)	16 (6.7)	240 (100)	
Education status				
10th pass	28 (96.5)	1 (3.4)	29 (100)	13.44 (0.001)*
12th pass	73 (82.02)	16 (17.9)	89 (100)	
Graduation and above	241 (94.1)	15 (5.9)	256 (100)	
Socio-economic status (as per modified Brahm Govind Prasad Scale)				
Upper class	309 (92.7)	24 (7.2)	333 (100)	7.06 (0.015)**
Upper-middle class and middle class	33 (80.5)	8 (19.5)	41 (100)	
Year of service				
<10 years	109 (93.2)	8 (6.8)	117 (100)	0.643 (0.423)*
>10 years	233 (90.6)	24 (9.4)	257 (100)	
Duty hours/week				
<48	110 (93.2)	8 (6.8)	118 (100)	1.60 (0.448)
49-72	163 (89.6)	19 (10.4)	182 (100)	
>72	69 (93.2)	5 (6.8)	74 (100)	
Duty hours/week				
<24 Hours	256 (92.8)	20 (7.2)	276 (100)	2.30 (0.129)
>24 Hours	86 (87.8)	12 (12.2)	98 (100)	
Holidays in a month				
<4 Days	275 (91.9)	24 (8.1)	299 (79.9)	0.534 (0.465)
>4 Days	67 (89.3)	8 (10.7)	75 (20.1)	

## Discussion

The present study found a high prevalence of occupational stress - 91% of study participants (54.3% for high and 37.2% for moderate stress) reported operational stress, and 54% of study participants (29% for moderate and 25% for high stress) reported organisational stress. The prevalence of operational stress as found in this study is comparable to that reported by Vivek et al. (2018) (90%), and higher than that reported by Furmeen et al. (2014) in Chitradurga, Karnataka (71%), and Nameirakpam et al. (2021) in Bishnupur District, Manipur (73%) [6,9,14]. The prevalence of organisational stress was lower when compared to that reported by Ragesh et al. (2017) (72%) and Vivek et al. (2018) (82%) [8,9]. The difference in findings could be due to the fact that the study by Vivek et al. (2018) included only female police personnel, and studies have shown prevalence to be higher among women. It may be due to the multiple roles that are expected by society and performed by females without adequate support. Different time periods and study settings can also lead to this difference. This study found that the prevalence of operational stress was higher than organizational stress; a similar finding was also observed by Vivek et al.(2018) in their study [9]. This is, however, in contrast to what few other studies have reported [8].

With regard to medical history, in the present study, 10.2% of the study participants reported having diabetes mellitus, and 14% of the participants reported having hypertension. These findings go along with the findings of other studies [4,15,16]. In the present study, around 11.2% and 22% of the participants gave a positive history of smoking and alcohol consumption, respectively, which is comparable to that reported by Abraham et al. (2019) (smokers=21%, alcohol consumers=31.2%) [4]. However, this might be underreported by the participant in lieu of social acceptance. Alcohol and tobacco use are the key unhealthy behaviours that impact health and work performance. In the present study, around one-fourth of the study participants reported difficulty sleeping. Vivek et al. (2018) in their study found that 76% of the participants were sleeping for less than six hours per day, while Galanis et al. (2019) found the mean hours of sleep that the participants get per day to be 7.1 (+1.2) hours [9,17]. Irregular shift work, night shifts, and extended working hours can result in sleep problems among police personnel. These findings are an eye-opener.

The important findings of the study are the leading stressors. The top five organisational stressors reported by the study participants are staff shortage, followed by lack of resources, excessive administrative duties, unequal sharing of work, lack of training, and feeling accountable for doing one's job. These stressors have also been highlighted by some of the previous studies as well [4,11,16]. The leading operational stressors reported are not having enough time available to spend with friends and family, managing social life outside work, finding time to stay in good physical condition, fatigue, and feeling like always being on the job, which have been highlighted as the major stressors in by few other studies [4,6,8,11].

While some Indian and international studies have found female participants to be more stressed than their counterparts, the present study has not found this to be the case in Delhi [14,18,19]. This can be due to differences in the socio-cultural environment in Delhi or different times and settings under which studies were conducted. The present study found a significant association between organisational stress and factors like the type of family, educational status, working for more than 72 hours per week, and service duration >10 years. Participants living in joint families and having educational qualifications of 10th or graduate and above had proportionately higher stress. A similar association was also reported by Selokar et al.(2011) in their study [18].

The current study found that those working for more than 72 hours per week perceived more organisational stress. Selokar et al. (2011) also reported a statistically significant association between stress and working hours; those working for >8 hours daily were found to have more stress than those working for <8 hours per day [18]. According to International Labor Organization (ILO) conventions, statutory normal working hours for various classes of workers have been reduced gradually from 48 hours to 40 hours across countries. In South Africa, working hours for police vary from eight hours for administrative duties to 12 hours for shift duties; however, working hours for police personnel have not been defined in India. Defined working hours per day are important for the physical and mental health of all working professionals, including the police. Staff shortage further leads to long working hours for the police personnel. The study found that those in service for more than 10 years perceived more organisational stress, as was also reported by Parsekar et al. (2015) in their study [16]. This could be due to the fact that fresh recruits have more enthusiasm for the job, and at that time, they usually have fewer responsibilities and are also more physically fit. With time, enthusiasm declines, and frustration with the job and stress aggravate, along with the physiological changes in the body. The present study found a statistically significant association between operational stress and educational status(p=0.001) and socio-economic status (p=0.015), which is in line with some other studies [6,18]. This can be due to the gap between expectations and satisfaction with different educational qualifications. Participants belonging to the upper class, as per the modified Brahm Govind Prasad scale, were found to have more operational stress. This can be due to the different nature of societal and economic pressures that come with the socio-economic status. The study found a higher prevalence of organisational stress among higher echelons than lower echelons (58% in Rank Strata 1 vs 50.3% in Rank Strata 2), although the association was not statistically significant. This can be due to the difference in roles and responsibilities across the echelons. Similarly to this, Singh et al. (2019) reported that inspectors had the highest level of stress (overall) [20]. The present study did not find any association of occupational stress (organisational and operational) with age and marital status, whereas a few studies have reported higher stress among married and 31-40 years of age group participants [14,18]. This could be attributed to different study settings.

Limitations

Firstly, as it was a cross-sectional study, causal relationships between stress and various factors couldn't be established. Secondly, the use of a self-administered questionnaire might have resulted in under- or over-reporting of stress. Thirdly, some of the factors might have been under-reported (including addiction history, medical history, family income, and perceived stress) because of social desirability. Fourthly, the current study could not assess levels of stress separately in all the various subsets of police personnel, like those involved in law and order management, criminal investigations, administrative duties, etc. These limitations highlight areas for improvement in future research, which could benefit from more granular subgroup analyses. 

## Conclusions

To conclude, the prevalence of occupational stress was high among the studied police personnel based on the Operational Police Stress Questionnaire and Organizational Police Stress Questionnaire. The prevalence of operational stress was found to be more than organisational stress. A significant number of participants reported having diabetes and hypertension, and gave a history of smoking and alcohol consumption. The top five organisational stressors reported by the study participants are staff shortage, lack of resources, excessive administrative duties, unequal sharing of work, lack of training, and feeling accountable for doing one's job, which were found to be the leading causes of organisational stress among the participants. Whereas, not having enough time available to spend with friends and family, managing social life outside work, finding time to stay in good physical condition, fatigue, and feeling like always being on job were the major operational stressors. These findings underscore the urgent need for comprehensive stress management and wellness interventions tailored to police personnel. 
